# A Natural Wish

**Published:** 1861-12

**Authors:** 


**A NATURAL WISH.**—It is very natural that we should wish the circulation of this JOURNAL largely extended. Has it ever occurred to our subscribers that it is their duty to do something toward this extension? If you have derived benefit from it, would it not be a kindly and neighborly act to induce your nearest friends to take it a year on trial? for it is very certain that by following its counsels, their health and happiness would be promoted and their lives extended for years.

HAVE YOU CHILDREN? You know how indifferent they are to your advice in too many cases; you know also that more importance is attached to what they see in print than to what you might say in regard to health. Would not the chances of their being benefited be greatly increased if you were to order the JOURNAL for each one of them? Their illness would be your greatest trouble, their death an irremediable grief; and yet thousands of lives are lost every year from inattention to the suggestions of one single article, to wit—about cooling off slowly after exercise. Many who have taken the JOURNAL, entire strangers to us, have written letters, or called in person, to express their obligations for the important and beneficial counsels found in our pages; and it is reasonable to infer that others taking it next year for the first time would derive similar benefits, both for themselves and for their children. We have no axes to grind, no medicines to sell, no patent contrivances to put in the market; but we do have a desire to promote the health and happiness of our kind, by the circulation of plain, practical truths in connection with human health; and we are no more under obligation to do this than you, reader! But it is too true that the friends of truth move with the snail, while the advocates of error vie with the hart and the hind in their efforts to circulate what is specious, false, and ruinous. He who lives wholly to himself and for himself is among the most contemptible of his kind, and never can be happy, never can be blest. They only are God-like, and akin to angels, who work for the good of others, and who delight in that work.

